# Streptococcal Toxic Shock Syndrome in a Child With Venous Malformation

**DOI:** 10.7759/cureus.21096

**Published:** 2022-01-10

**Authors:** Taro Yoshida, Yoshiko Asakura, Shoko Miura, Mikiya Endo, Manami Akasaka

**Affiliations:** 1 Pediatrics, Iwate Medical University Hospital, Morioka, JPN

**Keywords:** group a β-hemolytic streptococcus pyogenes, vascular malformation, venous malformation, cellulitis, streptococcal toxic shock syndrome

## Abstract

We report the case of a child with a venous malformation (VM), in whom streptococcal toxic shock syndrome (STSS) developed from cellulitis. A six-year-old boy with VM of the left lower limb had a fever and left lower limb pain since the afternoon of the day before admission. He presented with swelling, redness, heat, and tenderness on an area extending from the sole of the foot to the lower leg on the left side. Disturbance of consciousness gradually appeared, and he was admitted to the intensive care unit. We administered intravenous antibiotics and an immunoglobulin. On day two of hospitalization, group A hemolytic streptococci were detected in the blood culture. We managed the patient in coordination with a plastic surgeon for consideration of surgical interventions. The local findings subsequently improved to change the antibiotics promptly without debridement, and he was discharged after 14 days of antibiotic therapy. In this case, the VM may have contributed to the worsening of the infection. In children with VM, soft tissue inflammation with local pain and fever must be treated promptly, with the expectation of prompt surgical intervention, because the condition can progress to sepsis and necrotizing fasciitis.

## Introduction

Streptococcal toxic shock syndrome (STSS), caused mainly by group A β-hemolytic *Streptococcus pyogenes* (GAS), is a condition of septic shock that leads rapidly to multiple organ failure and disseminated intravascular coagulation. The prognosis at that point is poor; approximately 30% of those affected die [1].

In Japan, the first case was reported in 1993 [2] and then the National Institute of Infectious Diseases reports approximately 100 cases every year among adults and children [1]. The number of reports in children is extremely small, less than 10% that of cases in adults, but the number has been increasing in recent years [1]. STSS are triggered by infection with diabetes, cancers and immunosuppressive status in adults whereas infection with injury, chicken-pox and venous malformations (VM) may cause STSS in children [3].

We report the case of a child with VM of the left lower extremity in whom STSS developed as a result of cellulitis.

## Case presentation

**Figure 1:**
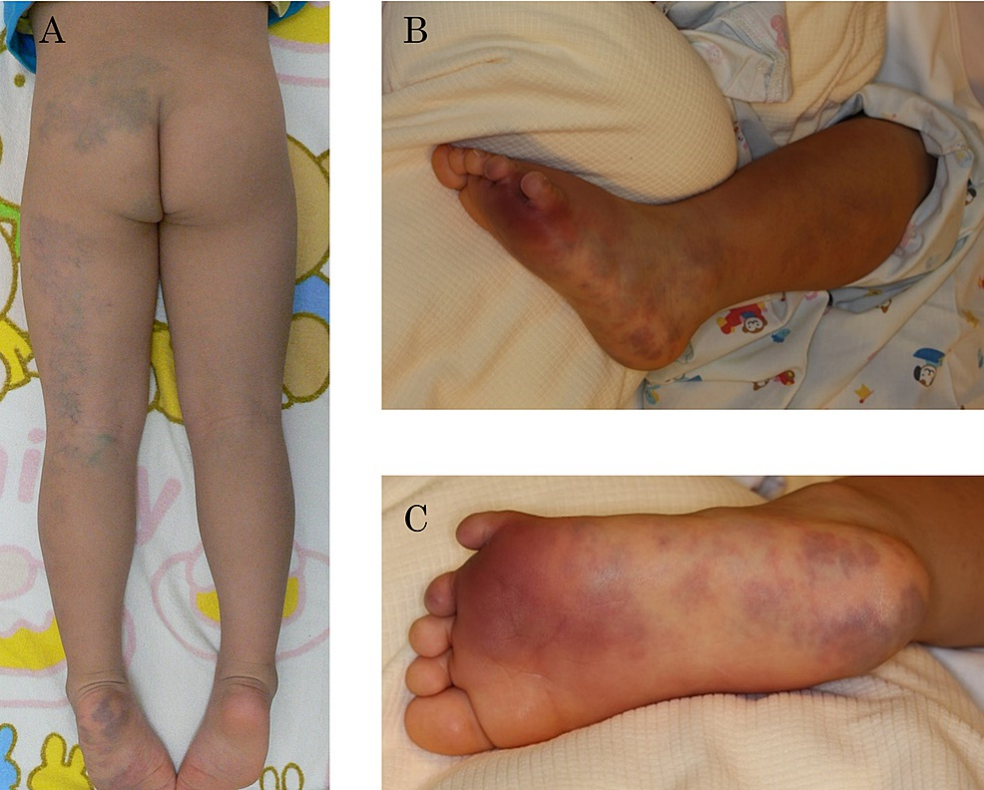
Cutaneous findings on the lower legs A. Findings before onset of streptococcal toxic shock syndrome (STSS). Multiple vascular lesions are present over the left buttock, thigh, lower leg, and sole. B. Cutaneous findings on the lower leg at onset. There is redness and swelling of the entire lower leg. C. Redness, heat, and swelling from the left fourth and fifth toes to the sole of the foot.

The patient was a six-year-old boy with no family history. Vascular lesion has been present on the left lower extremity since birth, and it has been monitored closely as a vascular malformation without therapy in the Department of Plastic Surgery at our hospital (Figure 1A). On the afternoon before admission, he developed fever and pain in the left lower extremity. When he visited our hospital, swelling, redness, and pain in the left lower extremity were observed, and consciousness disorder gradually appeared; he was therefore admitted to the intensive care unit.

His body temperature was 40.3°C; pulse, 220/min; blood pressure, 137/58 mm Hg; respiratory rate, 30/min; and oxygen saturation, 92% on room air. His level of consciousness was 10 on the Glasgow Coma Scale (E4V2M4). Pupillary light reflexes were rapid bilaterally, no peripheral coldness was observed in the other extremities, and capillary refill time was less than 2 s. Multiple vascular lesions were present in the area on the left buttock, the dorsal surface of the left thigh, the lower left leg, and the sole of the left foot. We observed marked redness, heat, and swelling from the left fourth and fifth toes to the sole of the foot (Figure 1B, 1C).

**Figure 2:**
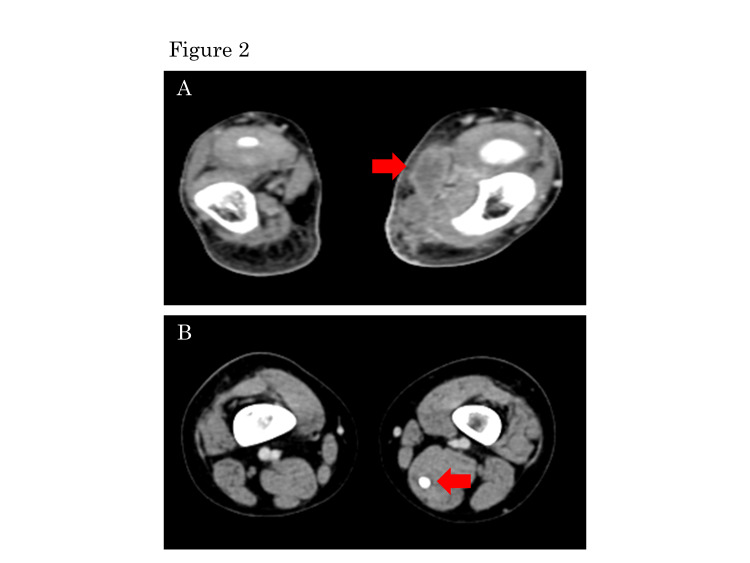
Contrast enhanced CT findings of the thigh at the onset of streptococcal toxic shock syndrome (STSS) A. Low-density areas with a contrasted capsule were seen in the soleus and crural muscles. B. Venous calculus were seen in the left semimembranosus muscle (arrow).

Hematobiochemical examination revealed an elevated white blood cell count (21,580/µL, with 95.1% neutrophils) and a C-reactive protein level of 7.5 mg/dL. Levels of muscle-derived enzymes were not elevated (creatine kinase, 64U/L). The platelet count was normal on admission but then dropped by more than 50% within 24 hours after admission; this development met the diagnostic criteria for disseminated intravascular coagulation. Cerebrospinal examination and head computed tomography (CT) yielded normal findings. Contrast medium-enhanced CT of the lower extremities showed low-density areas with strong contrast enhancement in the left leg muscles and irregular high-density areas in the left semimembranosus and gluteus maximus muscle (Figure 2A). Venous calculus was found in the left semimembranosus muscle (Figure 2B).

**Figure 3:**
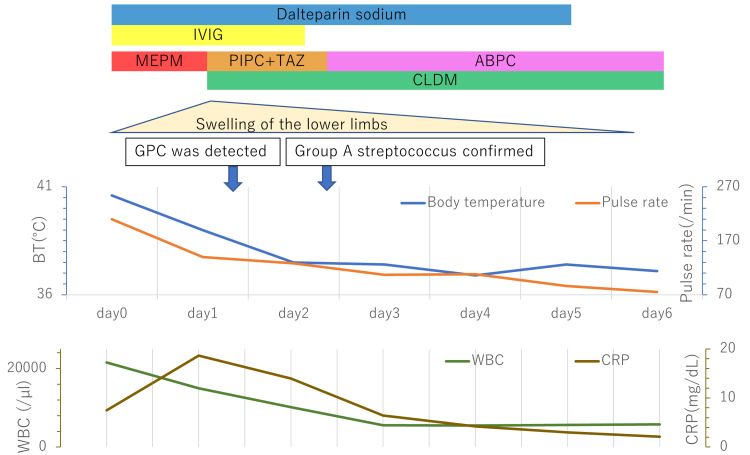
Clinical course after admission Abbreviation: ABPC, ampicillin; CLDM, clindamycin; CRP, c-reactive protein; IVIG, intravenous immunoglobulin; MEPM, meropenem; PIPC+TAZ, piperacillin/tazobactam; GPC, gram positive cocci; WBC, white blood cell

We administered meropenem, gamma globulin, and low-molecular-weight heparin immediately (Figure 3). Gram-positive cocci were detected in the blood cultures two days later, and we changed meropenem to piperacillin/tazobactam and added clindamycin. On day two of hospitalization, the pulse rate decreased to 110/min and the swelling and heat in the left lower extremity improved. The blood culture results confirmed GAS, and we changed piperacillin/tazobactam to ampicillin. After 14 days of intravenous antimicrobial therapy, the patient was discharged on day 17 after admission without surgical debridement.

Imaging evaluation for vascular malformations had not been performed in this patient until this admission. Contrast medium-enhanced CT of the lower extremities demonstrated venous calculi in the left semimembranosus muscle, and later, T2-weighted magnetic resonance images of the lower extremities revealed venous dilatation, extending from the left lower leg to the sole of the foot on images; therefore, VM was diagnosed.

## Discussion

In Japan, approximately 100 cases of STSS were traditionally reported per year [1], but these numbers are increasing since 2010. The number of pediatric cases is far lower than adult cases [4]. In our case, the patient developed STSS due to cellulitis with a VM.

Antimicrobial susceptibility tests of the GAS detected in this case revealed susceptibility to meropenem, clindamycin, and ampicillin, which may be one of the reasons for the favorable outcome. Clinicians must account for such antimicrobial susceptibility when selecting antibiotics. In the drug susceptibility test of 243 GAS strains from cases of STSS in Japan, all bacterial strains were susceptible to penicillin G, ampicillin, cefazolin, cefotaxime, meropenem, and linezolid, whereas clindamycin-resistant strains accounted for 11.5% of them [1].

Necrotizing fasciitis should be differentiated from cellulitis as a soft tissue infection caused by streptococci because surgical debridement is necessary to improve outcome, however the initial symptoms are nonspecific and often difficult to differentiate from those of cellulitis early stage. In STSS, the infection often spreads only to deep tissues in the early stage and skin changes are lacking; thus, it is not easy to diagnose early. The laboratory risk indicator for the necrotizing fasciitis (LRINEC) score is a useful tool for distinguishing necrotizing fasciitis from other soft tissue infections [5]. The indicators include total white cell count, hemoglobin, sodium, glucose and serum creatinine. The cut off value for the LRINEC score is 6 points. In our patient, the LRINEC score was 6 and the skin erythema tended to enlarge mildly over time; hence, we carefully managed the patient in consultation with a plastic surgeon. The local findings improved quickly; and, antibiotic therapy was continued.

The complication of infection is uncommon in VM, and the pathogenesis of infection in VM is not fully understood. As the blood flow in VMs is slow and the hypoplasia of blood vessels and abnormal structures have been shown to cause blood flow congestion [6], it may be associated with bacterial growth. The incidence of infections among patients with VM was investigated in 620 cases of low-flow vascular malformations [7]. Of the 21 patients with infections, 19 had lymphatic malformations and two had VM. Two patients with VM had local infections in the head and neck, and *Staphylococcus aureus* and *Streptococcus constellatus* were detected in wound and blood cultures, respectively. Infection recurred in 11 (52%) of the 21 patients. The incidence of recurrence was reported to be significantly lower among patients who received long-term antimicrobial therapy. In our patient, we administered antimicrobial agents intravenously for 14 days, and the infection did not recur.

A case of hemorrhagic shock that resulted from bleeding at the site of infection, in combination with septic shock from local infection, in a patient with VM has also been reported [8]. In that case, *Streptococcus dysgalactiae* subspecies *equisimilis* was detected in blood culture. That case suggests that VM may be a risk factor for the development of serious infections, other than STSS.

Two other cases of STSS in association with vascular malformations have been reported [3]. In these cases, soft tissue inflammation caused by intramuscular VM of the forearm led to the development of STSS. Their cases took 40 and 50 hours from the onset of initial symptoms to therapeutic intervention respectively, and both required surgical treatment, including amputation of the upper limb in one case. In our patient, in contrast, antimicrobial therapy was started approximately 24 hours after the onset of the symptoms, and the outcome suggests that the rapidity with which the patient was admitted to a medical institution and treatment was initiated was a factor in the favorable outcome.

Patients with VM often experience pain, and in an analysis of 2199 cases of VM in Japan, 45% were reported to be associated with pain [9]. Because the pain of VM, which is a frequent symptom, is difficult to distinguish from the pain associated with infection, affected patients may delay seeking medical attention. Therefore, patients with VM should be instructed to seek medical attention promptly, if even slight swelling, redness, or heat is observed in addition to pain especially in children. We should diagnose STSS early to improve their outcome.

## Conclusions

In case of children with VM, fever and soft tissue inflammation with local pain must be treated promptly. We should consult the patient in coordination with a plastic surgeon for consideration of surgical interventions, because the condition can progress to sepsis and necrotizing fasciitis. Our patient could recover with intravenous antimicrobial therapy successfully.
